# Supplementary material for: The association between cardiopulmonary exercise testing and postoperative outcomes in patients with lung cancer undergoing lung resection surgery: A systematic review and meta-analysis
Source: PLoS One. 2023 Dec 7;18(12):e0295430. doi: 10.1371/journal.pone.0295430 (PMC10703215; doi:10.1371/journal.pone.0295430)
Supplement: S2 Table — (PDF) [file pone.0295430.s002.pdf]

S2 Table - Search terms for electronic databases

Database	Search terms
PubMed	(((((preoperative care[MeSH Terms]) OR (preoperative period[MeSH Terms])) OR (risk assessment[MeSH Terms])) OR (preoperative*[Title/Abstract])) OR (pre-operative*[Title/Abstract])) OR (pre-surgery[Title/Abstract])<br>AND<br>((((((((((lung neoplasm/surgery[MeSH Terms]) OR (pneumonectomy[MeSH Terms])) OR (lung neoplasm[MeSH Terms])) OR (thoracic surgery[MeSH Terms])) OR (thoracic surgical procedures[MeSH Terms])) OR ("lung resect*"[Title/Abstract])) OR (“lung cancer”[Title/Abstract])) OR (“non-small cell lung cancer”[Title/Abstract])) OR ("lung cancer surgery"[Title/Abstract])) OR ("pulmonary surgical procedure"[Title/Abstract])) OR (lobectomy[Title/Abstract])) OR ("wedge resection"[Title/Abstract]))) NOT (cardiac surgery[MeSH Terms])<br>AND<br>((((((((((((((((((((exercise test*[MeSH Terms]) OR (oxygen consumption[MeSH Terms])) OR (physical fitness[MeSH Terms])) OR (anaerobic threshold[MeSH Terms])) OR ("exercise capacity"[Title/Abstract])) OR ("cardiopulmonary exercise"[Title/Abstract])) OR (CPET[Title/Abstract])) OR (CPEX[Title/Abstract])) OR (cpx[Title/Abstract])) OR (vo2[Title/Abstract])) OR (VO2max[Title/Abstract])) OR ("anaerobic threshold"[Title/Abstract])) OR (“minute ventilation”[Title/Abstract])) OR (“oxygen uptake”[Title/Abstract])) OR ("aerobic capacity"[Title/Abstract])) OR ("peak oxygen uptake"[Title/Abstract])) OR ("preoperative exercise"[Title/Abstract])) OR ("pre-operative exercise"[Title/Abstract])) OR ("cardiopulmonary exercise test*"[Title/Abstract])) OR (“cardiopulmonary stress test*”[Title/Abstract])) OR (“aerobic fitness”[Title/Abstract]))) NOT (prehabilitation[Title/Abstract])) NOT (rehabilitation[Title/Abstract])) NOT (training[Title/Abstract]))<br>AND<br>(((((((((((((postoperative complications/etiology[MeSH Terms]) OR (postoperative complications/physiopathology[MeSH Terms])) OR (postoperative complications/mortality[MeSH Terms])) OR ("postoperative outcomes"[Title/Abstract])) OR (post-operative[Title/Abstract])) OR (postoperative[Title/Abstract])) OR ("post-operative outcomes"[Title/Abstract])) OR (mortality[Title/Abstract])) OR ("survival analysis"[Title/Abstract])) OR (prognosis[Title/Abstract])) OR ("length of sty"[Title/Abstract])) OR (complications[Title/Abstract]))
CINAHL	preoperative OR pre-operative OR pre-surgery<br>AND<br>"lung resect*" OR “lung cancer” OR “non-small cell lung cancer” OR “lung cancer surgery” OR “pulmonary surgical procedure” OR lobectomy OR “wedge resection”<br>AND<br>"exercise capacity" OR "cardiopulmonary exercise" OR CPET OR CPEX OR cpx OR Vo2 OR VO2max OR "anaerobic threshold" OR “minute ventilation” OR “oxygen uptake” OR "aerobic capacity" OR "peak oxygen uptake" OR "preoperative exercise" OR "pre-operative exercise” OR "cardiopulmonary exercise test*" OR “cardiopulmonary stress test*” OR “aerobic fitness”<br>AND<br>"postoperative outcomes” OR post-operative OR postoperative OR "post-operative outcomes" OR mortality OR "survival analysis" OR prognosis OR "length of stay" OR complications<br>NOT<br>"cardiac surgery" OR prehabilitation OR rehabilitation OR training

Scopus	(TITLE-ABS-KEY (preoperative*) OR TITLE-ABS-KEY (pre-operative*) OR TITLE-ABS-KEY (pre-surgery))<br>AND<br>(TITLE-ABS-KEY ("lung resect*") OR TITLE-ABS-KEY ("lung cancer") OR TITLE-ABS-KEY ("non-small cell lung cancer") OR TITLE-ABS-KEY ("lung cancer surgery") OR TITLE-ABS-KEY ("pulmonary surgical procedure") OR TITLE-ABS-KEY (lobectomy) OR TITLE-ABS-KEY ("wedge resection"))<br>AND<br>(TITLE-ABS-KEY ("exercise capacity") OR TITLE-ABS-KEY ("cardiopulmonary exercise") OR TITLE-ABS-KEY (cpet) OR TITLE-ABS-KEY (cpex) OR TITLE-ABS-KEY (cpx) OR TITLE-ABS-KEY (vo2) OR TITLE-ABS-KEY (vo2max) OR TITLE-ABS-KEY ("anaerobic threshold") OR TITLE-ABS-KEY ("minute ventilation") OR TITLE-ABS-KEY ("oxygen uptake") OR TITLE-ABS-KEY ("aerobic capacity") OR TITLE-ABS-KEY ("peak oxygen uptake") OR TITLE-ABS-KEY ("preoperative exercise") OR TITLE-ABS-KEY ("pre-operative exercise") OR TITLE-ABS-KEY ("cardiopulmonary exercise test*") OR TITLE-ABS-KEY ("cardiopulmonary stress test*") OR TITLE-ABS-KEY ("aerobic fitness"))<br>AND<br>(TITLE-ABS-KEY ("postoperative outcomes") OR TITLE-ABS-KEY (post-operative) OR TITLE-ABS-KEY (postoperative) OR TITLE-ABS-KEY ("post-operative outcomes") OR TITLE-ABS-KEY (mortality) OR TITLE-ABS-KEY ("survival analysis") OR TITLE-ABS-KEY (prognosis) OR TITLE-ABS-KEY ("length of stay") OR TITLE-ABS-KEY (complications))
Cochrane	(preoperative*):ti,ab,kw OR (pre-operative*):ti,ab,kw OR (pre-surgery):ti,ab,kw<br>AND<br>("lung resection"):ti,ab,kw OR ("lung cancer"):ti,ab,kw OR ("non-small cell lung cancer"):ti,ab,kw OR ("lung cancer surgery"):ti,ab,kw OR ("pulmonary surgical procedure"):ti,ab,kw OR (lobectomy):ti,ab,kw OR ("wedge resection"):ti,ab,kw<br>AND<br>("exercise capacity"):ti,ab,kw OR ("cardiopulmonary exercise"):ti,ab,kw OR (CPET):ti,ab,kw OR (CPEX):ti,ab,kw OR (cpx):ti,ab,kw OR (Vo2):ti,ab,kw OR (VO2max):ti,ab,kw OR ("anaerobic threshold"):ti,ab,kw OR ("minute ventilation"):ti,ab,kw OR ("oxygen uptake"):ti,ab,kw OR ("aerobic capacity"):ti,ab,kw OR ("peak oxygen uptake"):ti,ab,kw OR ("preoperative exercise"):ti,ab,kw OR ("pre-operative exercise"):ti,ab,kw OR ("cardiopulmonary exercise test*"):ti,ab,kw OR ("cardiopulmonary stress test*"):ti,ab,kw OR ("aerobic fitness"):ti,ab,kw<br>AND<br>("postoperative outcomes"):ti,ab,kw OR (post-operative):ti,ab,kw OR (postoperative):ti,ab,kw OR ("post-operative outcomes"):ti,ab,kw OR (mortality):ti,ab,kw OR ("survival analysis"):ti,ab,kw OR (prognosis):ti,ab,kw OR ("length of stay"):ti,ab,kw OR (complications):ti,ab,kw<br>NOT<br>"cardiac surgery" OR prehabilitation OR rehabilitation OR training
