# Supplementary material for: The association between cardiopulmonary exercise testing and postoperative outcomes in patients with lung cancer undergoing lung resection surgery: A systematic review and meta-analysis
Source: PLoS One. 2023 Dec 7;18(12):e0295430. doi: 10.1371/journal.pone.0295430 (PMC10703215; doi:10.1371/journal.pone.0295430)
Supplement: S3 Table — (PDF) [file pone.0295430.s003.pdf]

S3 Table – Mortality data

**VO2 peak (mL/kg/min) and 30-day mortality**						
**Authors (Year)**	**Mortality**		**Number of patients**	**No mortality**		**Number of patients**
	Mean	SD		Mean	SD	
Bechard et al. (1987)	6.10	1.13	2	16.10	5.20	48
Brunelli et al. (2009)	12.30	3.10	6	16.00	3.80	198
Torchio et al. (1998)	17.20	1.50	5	19.10	3.10	140
Torchio et al. (2017)	16.90	2.40	11	19.10	3.40	252
**VO2 peak (mL/kg/min) and 60-day mortality**						
**Authors (Year)**	**Mortality**		**Number of patients**	**No mortality**		**Number of patients**
	Mean	SD		Mean	SD	
Olsen et al. (1989)	7.80	1.50	7	11.30	2.70	22
**VO2 peak (mL/kg/min) and 90-day mortality**						
**Authors (Year)**	**Mortality**		**Number of patients**	**No mortality**		**Number of patients**
	Mean	SD		Mean	SD	
Holden et al. (1992)	10.60	2.70	5	12.00	3.20	11
**VO2 peak (mL/kg/min) and unspecified time mortality**						
**Authors (Year)**	**Mortality**		**Number of patients**	**No mortality**		**Number of patients**
	Mean	SD		Mean	SD	
Win et al. (2005)	16.50	5.20	4	18.90	4.50	95

**VO2 peak (L/min) and 30-day mortality**						
**Authors (Year)**	**Mortality**		**Number of patients**	**No mortality**		**Number of patients**
	Mean	SD		Mean	SD	
Torchio et al. (1998)	1.03	0.19	5	1.40	0.33	140
Torchio et al. (2017)	1.04	0.16	11	1.40	0.33	252

**VO2 peak mL/kg/min percentage predicted (%) and 30-day mortality**						
**Authors (Year)**	**Mortality**		**Number of patients**	**No mortality**		**Number of patients**
	Mean	SD		Mean	SD	
Brunelli et al. (2009)	48.50	13.30	6	62.00	13.80	198
**VO2 peak mL/kg/min percentage predicted (%) and unspecified time mortality**						
**Authors (Year)**	**Mortality**		**Number of patients**	**No mortality**		**Number of patients**
	Mean	SD		Mean	SD	
Win et al. (2005)	61.30	15.10	4	89.50	31.10	95

VO2 peak L/min percentage predicted and 30-day mortality						
**Authors (Year)**	**Mortality**		**Number of patients**	**No mortality**		**Number of patients**
	Mean	SD		Mean	SD	
Torchio et al. (1998)	55.70	13.10	5	65.50	15.60	140
Torchio et al. (2017)	53.20	8.70	11	66.60	16.40	252

VO2 peak mL/min percentage predicted and 30-day mortality						
**Authors (Year)**	**Mortality**		**Number of patients**	**No mortality**		**Number of patients**
	Mean	SD		Mean	SD	
Larsen et al. (1997)	55.00	19.00	7	83.00	17.70	90

Load achieved (watts) and 30-day mortality						
**Authors (Year)**	**Mortality**		**Number of patients**	**No mortality**		**Number of patients**
	Mean	SD		Mean	SD	
Brunelli et al. (2009)	92.70	35.10	6	98.80	35.00	198
Larsen et al. (1997)	71.60	27.70	7	103.60	34.60	90
