# Supplementary material for: The association between cardiopulmonary exercise testing and postoperative outcomes in patients with lung cancer undergoing lung resection surgery: A systematic review and meta-analysis
Source: PLoS One. 2023 Dec 7;18(12):e0295430. doi: 10.1371/journal.pone.0295430 (PMC10703215; doi:10.1371/journal.pone.0295430)
Supplement: S4 Table — (PDF) [file pone.0295430.s004.pdf]

S4 Table – Morbidity data

VO2 peak (mL/kg/min) and in-hospital morbidity						
**Authors (Year)**	**Complications**		**Number of patients**	**No complications**		**Number of patients**
	Mean	SD		Mean	SD	
Campione et al. (2010)	11.28	3.34	19	11.07	3.20	80
Fang et al. (2013)	20.40	4.88	26	23.90	4.38	81
Licker et al. (2011)	13.00	4.40	46	16.90	5.20	164
Ribas et al. (1998)	17.10	4.30	31	16.10	3.70	34
Shafiek et al. (2016)	16.70	3.60	23	16.50	3.90	28
Fajardo et al. (2014)	18.85	3.83	72	18.78	4.42	11
Wang et al. (2000)	15.00	2.40	19	19.20	4.30	38
Yakal et al. (2018)	20.93	4.20	54	22.07	4.15	71
Win et al. (2005)	17.50	4.70	25	19.20	4.50	74
**VO2 peak (mL/kg/min) and 30-day morbidity**						
**Authors (Year)**	**Complications**		**Number of patients**	**No complications**		**Number of patients**
	Mean	SD		Mean	SD	
Bechard et al. (1987)	9.95	1.52	8	17.01	0.77	42
Brat et al. (2016)	9.60	2.90	56	10.70	3.00	20
Brunelli et al. (2009)	14.95	3.60	44	16.10	3.80	160
Brutsche et al. (2000)	17.90	5.40	31	22.10	5.40	94
Chouinard et al. (2022)	17.30	3.90	92	19.10	4.50	501
Epstein et al. (1993)	16.40	1.40	14	16.70	0.80	28
Loewen et al. (2007)	15.24	0.36	138	16.71	0.30	208
Mazur et al. (2022)	19.28	2.06	49	20.33	2.47	245
Pate et al. (1996)	14.10	2.60	7	13.67	1.90	5
Rodrigues et al. (2016)	15.65	1.13	18	16.48	0.76	32
Smith et al. (1984)	14.90	0.90	11	22.40	1.40	11
Torchio et al. (1998)	17.60	1.80	21	19.30	3.20	124
Torchio et al. (2017)	17.50	2.60	43	19.30	3.40	220
Matsuoka et al. (2004)	16.50	2.90	9	20.60	5.10	121
**VO2 peak (mL/kg/min) and unspecified time morbidity**						
**Authors (Year)**	**Complications**		**Number of patients**	**No complications**		**Number of patients**
	Mean	SD		Mean	SD	
Olsen et al. (1989)	10.10	2.50	13	11.50	2.00	16

VO2 peak L/min and in-hospital morbidity						
**Authors (Year)**	**Complications**		**Number of patients**	**No complications**		**Number of patients**
	Mean	SD		Mean	SD	
Shafiek et al. (2016)	1.14	0.26	23	1.23	0.36	28
Wang et al. (2000)	1.10	0.30	19	1.42	0.37	38
**VO2 peak L/min and 30-day morbidity**						
**Authors (Year)**	**Complications**		**Number of patients**	**No complications**		**Number of patients**
	Mean	SD		Mean	SD	
Brutsche et al. (2000)	1.32	0.41	31	1.59	0.42	94
Colman et al. (1982)	1.17	0.32	27	1.18	0.44	20
Epstein et al. (1993)	1.20	0.10	14	1.30	0.70	28
Larsen et al. (1997)	1.27	0.37	59	1.54	0.42	26
Rodrigues et al. (2016)	1.08	0.12	18	1.12	0.10	32
Smith et al. (1984)	1.00	0.06	11	1.59	0.14	11
Torchio et al. (1998)	1.30	0.30	21	1.40	0.30	124
Torchio et al. (2017)	1.20	0.30	43	1.40	0.30	220

AT (ml/kg/min) and all morbidity						
**Authors (Year)**	**Complications**		**Number of patients**	**No complications**		**Number of patients**
	Mean	SD		Mean	SD	
Brunelli et al. (2009)	9.60	1.90	44	10.10	3.90	160
Kasikcioglu et al. (2009)	14.30	2.40	18	16.20	3.50	31
Yakal et al. (2018)	14.83	3.38	54	15.26	3.26	71

VO2 peak mL/kg/min percentage predicted (%) and in-hospital morbidity						
**Authors (Year)**	**Complications**		**Number of patients**	**No complications**		**Number of patients**
	Mean	SD		Mean	SD	
Fang et al. (2013)	63.70	19.04	26	79.00	11.60	81
Licker et al. (2011)	50.00	17.00	46	60.00	17.00	164
Ribas et al. (1998)	83.00	33.00	31	74.00	22.00	34
Shafiek et al. (2016)	75.10	20.60	23	74.80	16.50	28
Wang et al. (2000)	70.00	14.00	19	76.00	15.00	38
**VO2 peak mL/kg/min percentage predicted (%) and 30-day morbidity**						
**Authors (Year)**	**Complications**		**Number of patients**	**No complications**		**Number of patients**
	Mean	SD		Mean	SD	

Brunelli et al. (2009)	59.10	14.40	44	67.40	13.70	160
Brutsche et al. (2000)	80.00	17.00	31	99.00	21.00	94
Chouinard et al. (2022)	94.50	26.00	92	101.70	28.60	501
Loewen et al. (2007)	66.55	1.75	138	75.34	1.43	208
Rodrigues et al. (2016)	60.00	4.25	18	69.50	6.25	32
Smith et al. (1984)	55.20	3.00	11	73.70	5.40	11
**VO2 peak mL/kg/min percentage predicted (%) and unspecified time morbidity**						
**Authors (Year)**	**Complications**		**Number of patients**	**No complications**		**Number of patients**
	Mean	SD		Mean	SD	
Villani et al. (2003)	71.00	1.80	44	75.60	1.30	106
Win et al. (2005)	70.70	19.50	25	94.30	32.10	74

**VE/VCO2 slope and 30-day morbidity**						
**Authors (Year)**	**Complications**		**Number of patients**	**No complications**		**Number of patients**
	Mean	SD		Mean	SD	
Brat et al. (2016)	38.80	6.40	56	35.40	6.10	20
Chouinard et al. (2022)	38.80	7.50	92	36.30	7.10	501
Shafiek et al. (2016)	40.80	6.10	23	38.80	8.70	28
Torchio et al. (1998)	35.00	8.30	21	32.00	5.88	124
Torchio et al. (2017)	33.10	7.70	43	32.70	5.70	220
Yakal et al. (2018)	34.84	7.36	54	36.02	7.55	71
Mazur et al. (3022)	31.50	2.89	49	29.00	2.32	245

**Load achieved (watts) and all morbidity**						
**Authors (Year)**	**Complications**		**Number of patients**	**No complications**		**Number of patients**
	Mean	SD		Mean	SD	
Brunelli et al. (2009)	100.9	33.00	44	98.00	35.50	160
Brutsche et al. (2000)	122.00	30.00	31	133.00	34.00	94
Chouinard et al. (2022)	94.30	30.80	92	101.90	94.30	501
Epstein et al. (1993)	90.00	10.00	14	86.00	8.00	28
Licker et al. (2011)	90.00	30.00	46	109.00	34.00	164
Larsen et al. (1997)	92.40	33.00	59	116.90	33.00	26
Rodrigues et al. (2016)	73.10	7.43	18	71.70	7.70	32
Shafiek et al. (2016)	77.70	21.80	23	78.90	26.70	28
Wang et al. (2000)	90.00	30.00	19	117.00	29.00	38
