# Supplementary material for: The association between cardiopulmonary exercise testing and postoperative outcomes in patients with lung cancer undergoing lung resection surgery: A systematic review and meta-analysis
Source: PLoS One. 2023 Dec 7;18(12):e0295430. doi: 10.1371/journal.pone.0295430 (PMC10703215; doi:10.1371/journal.pone.0295430)
Supplement: Attachment — Submitted filename: Response to Reviewers.docx [file pone.0295430.s005.docx]

To the Editor in Chief (PLoS One) & Dr Benjamin Hunter

We would like to thank you for taking the time to review our paper. Your input was so helpful to strengthen the paper and make it more clinically useful. Please see all revisions below. We hope that we have covered all the points sufficiently and look forward to any further feedback.

Many thanks

Dr. Nabeela Arbee-Kalidas, Dr. Jacob Moutlana, Dr. Yoshan Moodley, Dr. Moses Kebalepile & Prof. Palesa Motshabi Chakane

**Reviewers comments**

| **Major revisions** | | |
| --- | --- | --- |
| **Reviewer’s comments** | **Authors’ comments** | **Revision** |
| The rationale for the review could be strengthened. For instance, why are ventilatory equivalents and demarcations of exercise intensity domains of interest to practitioners? A summary of the importance of these factors in the context of existing knowledge could strengthen the rationale. This is a requirement of the PRISMA guidelines. | Thank you for the detailed review. Noted. | Please see revised introduction which includes more detail of the existing literature to strengthen the rationale. |
| In the discussion, the implications and explanation of why CPET variables are of use to clinicians is sometimes lacking in appropriate detail. As such, the discussion is quite brief. It would be good to have a more detailed discussion on the utility and significance of these parameters on postoperative outcomes in patients. Further, the heterogeneity of the literature should be discussed in more detail. | Thank you for the input. | Please see completely revised discussion section written in more detail. |

| **Abstract** | | |
| --- | --- | --- |
| **Reviewer’s comments** | **Authors’ comments** | **Revision** |
| Pg 2, Ln 27: 37 should be Thirty-seven. | Noted with thanks | 37 changed to Thirty-seven. |
| Pg 2, Ln 27: How many had low bias? | 28 | Sentence changed to read “Twenty-eight studies had low risk of bias”. |
| Pg 2, Ln 27: VO2 should have a diacritic mark over the V. Please check throughout. | Noted, thank you | VO2 peak and VE/VCO2 changed to $\dot{V}$ throughout. |
| Pg 2, Ln 41: This sentence structure is difficult to follow, suggest rewording. | Agreed | Sentence altered to read “This study found that patients with a lower $\dot{V}$E/$\dot{V}$CO_2_ and patients with a higher $\dot{V}$O_2_ peak value (measured or as a percentage of postoperative predicted) had better postoperative outcomes following lung resection surgery for lung cancer.” |
| Pg 2, Ln 38: how was heterogeneity defined? |  | Added to Ln 38, pg 2. . I^2^ > 40% indicated a high level of heterogeneity. |

| **Introduction** | | |
| --- | --- | --- |
| **Reviewer’s comments** | **Authors’ comments** | **Revision** |
| Pg 4, Ln 48: First two paragraphs could be grouped together. |  | First two paragraphs grouped together. |
| Pg 4, Ln 58: It may be nice to offer some limitations of the use of CPET to determine fitness for surgery. |  | Limitations added Pg 4 Ln 79-83. |
| Pg 4, Ln 62: Delete “had” |  | “had” deleted. |
| Pg 5, Ln 76: How are you defining “anaerobic threshold”? Is it just oxygen consumption at AT or workload as well? | We defined AT as it was described in the studies included in the meta-analysis (Yakal & Kasikcioglu) i.e. oxygen consumption at anaerobic threshold as measured by the V-slope method. Workload at AT was not used. | - |
| Pg 5, Ln 80-83: This is too much like your objectives, suggest deletion or modification. | Noted, with thanks. | Paragraph deleted. |

| **Methods** | | |
| --- | --- | --- |
| **Reviewer’s comments** | **Authors’ comments** | **Revision** |
| Pg 5, Ln 92: Full stop missing after reference. | Noted with thanks. | Full stop added. |
| Pg 6, Ln 99-107: Missing information about how studies were grouped for the synthesis. | Mentioned in a different section entitled “Summary measures and synthesis of results” on Pg 8 Ln 149: “For the analysis, the studies were grouped based on outcomes.” | - |
| Pg 7, Ln 118-122: What happened in case of conflicts between authors and how were they settled? | Apologies for not explaining that in sufficient detail. | Sentence now reads “Disagreements were resolved through discussion with a third reviewer (PM).” |
| Pg 7, Ln 125-135: Workload is not given here. A few studies use treadmills for CPET, is end speed included or was it excluded from analysis? If excluded, a rationale should be provided. | Of the 6 studies that used treadmills, none commented on end-speed hence could not be included.  Work load, VO2 peak, VE/VCO2 and all other CPET variables that were commented on from each paper and used in correlation analysis with postoperative outcomes were recorded by the authors. | Sentence now reads “CPET: type of CPET performed, if CPET was used in preoperative risk stratification i.e. blinding and all CPET variables used in the correlation analyses for each paper ($\dot{V}$O_2_ peak measured and percentage of predicted, $\dot{V}$E/$\dot{V}$CO_2,_ AT, peak exercise load)” |
| Pg 8, Ln 150: Outcomes used for grouping could be given here for clarity. |  | Sentence now reads “Outcomes: postoperative morbidity (in-hospital, 30-day, 60-day) and mortality (in-hospital, 30-day, 60-day, 90-day, ≥ 2 years)” |

| **Results** | | |
| --- | --- | --- |
| **Reviewer’s comments** | **Authors’ comments** | **Revision** |
| Pg 10, Ln 189: Sex or gender? Be consistent (see below). | Noted, with thanks. Sex is the preferred word. | Standardised to sex, male and female throughout. |
| Pg 11, Table 2: If using “gender”, the terms “men” and “women” should be used. If “sex”, your current use of male and female is fine. Where information is not given, e.g., the exercise modality for Begum et al., this could be made clearer to the reader. | The hyphen was used in the table to indicate that the information was not recorded. | The caption below the table now reads “hyphen (-) – missing information” |
| Pg 15, Ln 275: This is the first time you mention studies being excluded from the MA due to heterogeneity. Was this decided a priori? This should be mentioned in your methods. | Apologies, the word heterogeneity was used incorrectly here. The studies were not excluded due to heterogeneity. They could not be included in a meta-analysis because they did not present mean values like the rest of the studies did. They presented the data in a unique format so no pooling could be done. | Included the exclusion in Pg 8 Ln 186 “Studies presenting high variability of data type or format were analysed descriptively.” Also mentioned in Pg 9 Ln 214 “Eight studies were not included in the meta-analysis but were described as they were presented in the form of categories with no access to raw data and an inability to pool the data.” And Pg 15 Ln 407 |

| **Discussion** | | |
| --- | --- | --- |
| **Reviewer’s comments** | **Authors’ comments** | **Revision** |
| Ln 16, Pg 290-291: Suggest rewording the sentence beginning “VE/VCO2”, as it doesn’t necessarily reflect the minute ventilation required to eliminate CO2, it’s just the ratio of minute ventilation to carbon dioxide production. This measure is not a definitive indicator of ventilatory inefficiency. This should be expanded on. | Thank you. | Revised entire discussion to clarify this in more detail. |
| Pg 16, Ln 300: Current guidelines should be referenced here. | Noted, thank you. | Reference for BTS guidelines added. |
| Pg 17, Ln 304: This sentence could be more specific. Is this end stage exercise load? | Noted. It is load achieved at peak exercise i.e. the highest load in watts that the patient was able to cycle against. | Sentence changed to read “Our study did not find a strong association between peak exercise load achieved and mortality or morbidity. Benzo et al. found a significantly higher peak exercise load achieved in patients without postoperative pulmonary complications.” |
| Pg 17, 311-313: Reference needed here. |  | Reference added by Levett et al. “Perioperative cardiopulmonary exercise testing (CPET): consensus clinical guidelines on indications, organization, conduct, and physiological interpretation.” |
| Pg 17, 315: Reference needed here. |  | Reference added “ BTS guidelines: guidelines on the selection of patients with lung cancer for surgery.” |
| Pg 17, 315: This section is surprising, given it is the first time methods other than CPET are discussed. Unsure of the relevance of this. | Agreed, not necessary to introduce new and vague information in the last paragraph. It is out of the scope of this paper to go into every exercise test in detail. | Paragraph deleted. |
| Pg 18, Pg 329: A more detailed appraisal of the heterogeneity would be welcomed somewhere in the discussion. This is an important finding, and one that doesn’t seem to be explained in sufficient detail. | Thank you, noted. | Added some more detail regarding the heterogeneity in the limitations section Pg 19. |

| **Conclusion** | | |
| --- | --- | --- |
| **Reviewer’s comments** | **Authors’ comments** | **Revision** |
| Pg 19, Ln 354: Although this is a reasonable conclusion to draw, the significance of these variables are not clearly highlighted in the discussion. | Noted, with thanks. Hopefully the newly revised discussion addresses this. | - |

**Journal requirements**

| **Editors comments** | **Authors’ comments** | **Revision** |
| --- | --- | --- |
| Please ensure that your manuscript meets PLOS ONE's style requirements, including those for file naming. |  | Changes made to formatting, cover page and file naming. |
| In your Data Availability statement, you have not specified where the minimal data set underlying the results described in your manuscript can be found. | Noted. | All data has been made available in the supporting information. |
| Please include captions for your Supporting Information files at the end of your manuscript, and update any in-text citations to match accordingly | Noted with thanks. | Added. |
| We note that this manuscript is a systematic review or meta-analysis; our author guidelines therefore require that you use PRISMA guidance to help improve reporting quality of this type of study | Thank you. | Added as supporting information. |
| Please review your reference list to ensure that it is complete and correct. | Noted. | EndNote was used for all references. Vancouver format. All references checked. No included papers have been retracted as per EndNote, PubMed & Google Scholar search. |
