# Supplementary material for: The association between cardiopulmonary exercise testing and postoperative outcomes in patients with lung cancer undergoing lung resection surgery: A systematic review and meta-analysis
Source: PLoS One. 2023 Dec 7;18(12):e0295430. doi: 10.1371/journal.pone.0295430 (PMC10703215; doi:10.1371/journal.pone.0295430)
Supplement: Attachment — Submitted filename: Response to Reviewers.docx [file pone.0295430.s006.docx]

To the Editor in Chief (PLoS One) & the reviewers

We would like to thank you again for taking the time to review our paper. Please see all revisions below. We hope that we have covered all the points sufficiently and look forward to any further feedback.

Many thanks

Dr. Nabeela Arbee-Kalidas, Dr. Jacob Moutlana, Dr. Yoshan Moodley, Dr. Moses Kebalepile & Prof. Palesa Motshabi Chakane

**Reviewer 1 comments**

| **Reviewer’s comments** | **Revision** |
| --- | --- |
| Ln 78: Sentence beginning “Historically…” requires a reference. | Reference added, article by Older & Hall. |
| Ln 95: “< 34” needs units. | $\dot{V}$E/$\dot{V}$CO_2_ is not usually recorded as having units because it is a ratio. |
| Ln 239: I think “peak” has been accidentally deleted? Please check this. | Corrected. |
| Ln 272: “Figure 54” should be changed to “Figure 5”. | Noted. |
| Ln 275: Same as Ln 239. | Corrected. |
| Ln 363: Unsure of the wording “non-meta-analysed”, but this could just be down to personal preference! | The different cut-off values presented in the descriptive analysis (Table 3) need further validation. |
| Ln 392: This is an interesting point |  |

**Reviewer 2 comments**

| **Abstract** |  |
| --- | --- |
| **Reviewer’s comments** | **Revision** |
| Integrate first two sentences of the background section into one (lines 17 – 19) | Done. |
| Add definition of VO2 peak (in line 30). Indicate that VO2 peak is (one of the outcomes) that can be derived from CPED, and indeed the measure of CRF. | Added. Sentence reads “$\dot{V}$O_2_ peak is the oxygen consumption at peak exercise and serves as a marker of cardiopulmonary fitness.” |
| Line 38 – is this sentence on heterogeneity not already reported in methods? | The methods section explains what statistical value was used to define heterogeneity in the analysis. The results sections explains that heterogeneity was found in the literature. |
| Line 40 – higher VO2 peak, not better. Then, I find the expression better morbidity and mortality somewhat strange. Better prognosis? Reduced post-operative morbidity or mortality? | Wording changed. Sentence reads “Cardiopulmonary fitness as evidenced by higher peak oxygen consumption is associated with estimates of decreased morbidity and mortality. Ventilatory efficiency however, was associated with decreased postoperative morbidity but not with improved survival.” |

| **Introduction** |  |
| --- | --- |
| **Reviewer’s comments** | **Revision** |
| In line 73-75, you highlighted the difference between Benzo et al meta-analysis, and this current work. Would you be able to elaborate on why the observed effect in the general populations (lower VO2peak increases risk complications following pulmonary surgery) may be different in a population with lung cancer? | Benzo et al. included some patients undergoing lung resection surgery for infective and non-malignant reasons such as emphysematous bullae etc. thereby comparing non-benign to benign patients in the same meta-analysis which limits its applicability. Sentence now reads “The cardiopulmonary limitations in patients with lung cancer could be attributed to the multisystemic effects of the cancer such as global inflammation, sarcopenia, anaemia, fatigue and the side effects of chemotherapy or radiation therapy which patients with benign conditions may not experience.” |
| A minor point in Line 61 – when you state: the potential for incorrect display of graphical data what are you referring to? I have looked at your reference 8 and seem to be unable to locate any further info. | Inserted another reference by Neder et al. explaining the pitfall more accurately. When performing CPET, the practitioner usually examines the typical 9 panel plot which may not be appropriate for that particular patient/pathology. You may need to focus on the dynamic relationships more likely to expose the patterns of dysfunction rather than the standard plot. |
| Line 66 and throughout – appreciate AT is still the preferred term, but I would suggest amending the term anaerobic threshold, and refer to gas exchange threshold or lactate threshold instead | The term anaerobic threshold will remain, as this is the term used in the current CPET guidelines by Levett et al. “Perioperative cardiopulmonary exercise testing (CPET): consensus clinical guidelines on indications, organization, conduct, and physiological interpretation.” |
| Also in Line 360-362, that view of AT as the point where aerobic metabolism switches to ‘anaerobic’ metabolism is not generally accepted, and has been contested. | There is significant controversy surrounding it. The definition now reads the “The AT is the point above which lactate begins to increase during incremental exercise which indicates increased glycolysis”. This is the definition used in the guidelines mentioned above. |

| **Methods** |  |
| --- | --- |
| **Reviewer’s comments** | **Revision** |
| Line 115: did you consider whether standard care included exercise recommendations (that is, not a structured exercise programme but the recommendation to stay active?). | It was not considered in the initial methods however having read through all included papers, none of them make mention of encouraging an active lifestyle or promoting exercise in a non-structured way. |

| **Results** |  |
| --- | --- |
| **Reviewer’s comments** | **Revision** |
| As it reads, the numbers don’t quite work in Line 184-5: There were 1096 articles screened. Then - from the titles and abstracts, 1041 were excluded for not meeting the inclusion criteria. A total of 47 studies warranted a full-text review. But 1096 – 1041 is 55, not 47? Please add here that for a further 8 studies full-text article was not found. Also, are these the same 8 studies you describe in lines 189-191? Just needs some clarification. | No English full text could be found for the 8 studies that were “unaccounted for” in the text. We had displayed it on the PRISMA diagram but now have included it in the text for clarity. “A total of 55 studies warranted a full-text review however no full English text could be located for 8 studies. Of the 47 full-texts found, ten studies…” |
| The results may be a bit more clear when it comes to reporting VO2peak (mL/kg/min) – and other physiological parameters, vs reporting the same parameter (e.g. VO2 peak) as a % of predicted. How were these values predicted, as there are many equations that predict for eg. Based on a number of variables. Also, was this prediction done by the authors of the current study, or was it reported in every study? | The $\dot{V}$O_2_ peak as a percentage of predicted described in the methodology of the papers say that it is the percentage of the actual $\dot{V}$O_2_ peak that the patient achieves compared to the expected $\dot{V}$O_2_ peak for a healthy patient of the same sex and age. This was not calculated by the authors and was taken from the individual papers verbatim as they had commented on them. There are registries available with expected normal values eg. <https://bmjopen.bmj.com/content/8/3/e018697> however it does depend on the country you are in and the software used. Mentioned as a limitation.  VO2 peak and VO2 peak as a percentage of predicted have been split into separate paragraphs. |
| Line 243 & 274 – these lack of associations/differences may in part be explained by different protocols. A very steep (e.g. 15-20 W per min) may allow participants compared to a shallower protocol, of 5-10 W per min increases. Just consider this in your results and analyses (and interpretation – line 354). Perhaps the same could be said about AT. There are various protocols to estimate AT (or GET / LT). I am assuming in this context, these estimations are derived from a software (e.g. estimate AT via the v-slope method), but there is likely some variability and error in estimates, and differences between labs, equipment, protocols, etc. that may confound the results you are reporting herein. | Noted, this has been added to the discussion and limitations section. “Yakal et al. used the Bruce protocol which is a much steeper, faster ramp than the Naughton protocol used by Kasikcioglu et al. and this could mean patients with severe cardiopulmonary limitations in the study by Yakal et al. could find attaining the AT more challenging.” |

| **Discussion** |  |
| --- | --- |
| **Reviewer’s comments** | **Revision** |
| Perhaps the authors could consider adding CP as a (plausible marker). | We are not clear on what CP is? |
| I feel the discussion remains too generic, and there are some missed opportunities. Please do try and direct the discussion a bit more towards your own results. For example, in the second / third paragraph of the discussion, you report the threshold of 15 mL for a VO2peak – is that value still applicable considering the results reported in this meta-analysis? Should be revisited? The discussion reads a bit like a review/introduction. | Revised discussion section to be more specific. Confirmed that the authors do agree with the current cut-off of 15mL/kg/min for postoperative mortality, but not for morbidity and added some recommendations. Figures 4 and 10 have been added to indicate odds ratios. |
| Line 326/7 – clearly indicate here again what those cut-off values are. | Noted, corrected. |
| I think you should emphasise, more clearly, what the results of this meta-analysis. For example, the current guidelines was for VO2 peak to reach 15 mL, is that still the case after this study? | Newly revised discussion section addresses this. |

**Journal requirements**

| **Editors comments** | **Revision** |
| --- | --- |
| Please review your reference list to ensure that it is complete and correct. | All references re-checked. No included papers have been retracted as per EndNote, PubMed & Google Scholar search. |
